# Implication of the Annexin 1/FPR axis in leishmanial exosome-mediated *Leishmania major* skin hyperpathogenesis

**DOI:** 10.3389/fimmu.2024.1436151

**Published:** 2024-07-15

**Authors:** Alonso da Silva Lira Filho, Andrea Lafleur, Fernando Alvarez, Ciriaco A. Piccirillo, Martin Olivier

**Affiliations:** ^1^ Department of Microbiology and Immunology, McGill University, Montréal, QC, Canada; ^2^ Infectious Diseases and Immunity in Global Health Program, Research Institute of the McGill University Health Centre, Montréal, QC, Canada

**Keywords:** *Leishmania*, cutaneous leishmaniasis, extracellular vesicles, exosomes, Annexin A1, FPR2

## Abstract

**Introduction:**

Exosomes produced by the protozoan parasite *Leishmania* (LeishEXO) are well-established drivers of virulence, though mechanisms underlying their exacerbation of experimental leishmaniasis remain elusive. Expression of Annexin A1 (ANXA1), a protein implicated in exosome-mediated pathologies and viral internalization, has been shown to correlate with cutaneous leishmaniasis severity. Given ANXA1’s regulation of myeloid cells – the canonical hosts for *Leishmania* – we studied the potential role of ANXA1 and its receptors FPR1/2 in exerting LeishEXO’s effects.

**Methods:**

Murine and *in vitro* ANXA1^-/-^ models were used to study the generation of protective T_H_1 responses during experimental *L. major* infection with and without LeishEXO. Recruitment of inflammatory cells was assessed using a peritoneal cell recruitment assay and immunophenotyping, and production of inflammatory mediators was measured using a cytokine and chemokine array. Treatment of experimental models with FPR2 antagonist WRW4 and FPR1/2 agonist WKYMVm was used to delineate the role of the FPR/ANXA1 axis in LeishEXO-mediated hyperpathogenesis.

**Results:**

We established that ANXA1 deficiency prohibits LeishEXO-mediated pathogenesis and myeloid cell infection, with minimal alterations to adaptive and innate immune phenotypes. FPR2 blockade with WRW4 similarly inhibited leishmanial hyperpathogenesis, while direct activation of FPRs with WKYMVm enhanced infection and recapitulated the LeishEXO-mediated phenotype. This research describes LeishEXO’s utilization of the ANXA1/FPR axis to facilitate parasitic internalization and pathogenesis, which may be leveraged in the development of therapeutics for leishmaniasis.

## Introduction

Leishmaniases are an array of cutaneous and visceral pathologies caused by the protozoan parasite *Leishmania*, responsible for over 2 million new infections and 30,000 deaths annually (1). Infection typically occurs when a phlebotomine sandfly bearing the parasite takes a bloodmeal from a mammalian host, inoculating *Leishmania* into the host dermis (2). Parasites are then rapidly internalized by inflammatory myeloid cells recruited to the site of infection – most notably macrophages and neutrophils (2). While macrophages act as the canonical host cell for *Leishmania*, infected neutrophils become apoptotic before being, themselves, engulfed by macrophages, allowing the parasite to maximize its establishment through a “Trojan Horse” mechanism (2).

To ensure parasitic persistence, *Leishmania* promotes macrophage dysfunction by altering key signaling pathways and impairing the host cell’s ability to mount an effective parasitotoxic response (3, 4). Our previous research has established that critical leishmanial virulence factors, such as the surface metalloprotease GP63, are packaged within extracellular vesicles (EVs) which can, in turn, directly abrogate macrophage function and pre-emptively produce a favorable environment for parasitic uptake and persistence (3). Exosomes, which are ubiquitously-produced small EVs containing biologically active cargo (including proteins, nucleic acids, and lipids), have been the focus of numerous studies due to their implication in intercellular communication, and roles in the modulation of immunity, oncogenesis, and the immunopathogenesis of infectious disease (5–9).

We previously reported that *Leishmania* produces exosomes in the midgut of its sandfly vector, which are co-inoculated with parasites during the sandfly’s bloodmeal, in turn exerting an immunomodulatory effect (10). In fact, leishmanial exosomes isolated from *in vitro* cultures are as effective at modulating early macrophage and host inflammatory responses as whole *Leishmania* parasites (3, 11, 12). Furthermore, *Leishmania* EVs exacerbate cutaneous leishmaniasis lesions by inducing the overproduction of the inflammatory cytokine IL-23/IL-17, leading to the recruitment of neutrophils and pathogenic hyperinflammation (10, 11). However, while it is well-established that leishmanial exosomes are crucial modulators of macrophage function, potentiators of *Leishmania* infection, and drivers of immunopathogenesis, mechanisms underlying their effects remain elusive (3, 4, 10, 12).

The annexin-family protein Annexin A1 (ANXA1) has been implicated in EV-mediated exacerbation of non-communicable disease, including in the promotion of malignant cell proliferation and development of cardiovascular microcalcifications, notably through direct interaction with exosomal lipids and phospholipids, as well as through the stimulation of the downstream N-formyl peptide receptors (FPR) 1 and 2 (13–16). ANXA1 also has an established role in infectious disease, capable of facilitating viral entry into host cells (17). While ANXA1 has previously been reported to play a role in the regulation of myeloid cells at early time points following *Leishmania* infection, and expression levels of the protein have been shown to correlate with the severity of the histopathological features of cutaneous leishmaniasis, the effect of leishmanial exosome/ANXA1 interaction has yet to be studied (18, 19). Herein, we hypothesized that ANXA1 could be implicated in cellular events underlying leishmanial exosome-mediated exacerbation of cutaneous leishmaniasis. To study this, we utilized *in vivo*, *ex vivo*, and *in vitro* studies to provide evidence that leishmanial exosomes exploit the ANXA1/FPR axis to enhance myeloid cell invasion by *Leishmania* in the early stages of infection, driving immunopathogenesis. This research uncovers a novel role for this immunomodulatory mechanism, which may be leveraged in the development of therapeutics for leishmaniasis.

## Materials and methods

### Parasite culture

*Leishmania major* parasites used in the study were NIH S (MHOM/SN/74/Seidman) clone A2. Parasites were cultured at 25 ˚C, 5% CO_2_ in Schneider’s Drosophila Medium (SDM) supplemented with 10% heat-inactivated fetal bovine serum (FBS; Wisent, St-Bruno, QC, Canada), and 5 mg/mL Hemin. Cultures of promastigotes were passaged every 3–4 days to maintain logarithmic growth or were grown to stationary phase (day 6–8 post-passage) before being used in infections.

### Extraction of *L. major*-derived vesicles

To extract *L. major* exosomes/extracellular vesicles (LeishEXO), 800 mL of late log phase parasite culture (1–4 × 10^8^ parasites/mL) was centrifuged at 300 x g and pelleted. Parasites were then washed 3 times with PBS to remove dead cells and debris using centrifugation at 300 x g. Parasites were resuspended in FBS-free RPMI 1640 medium without phenol-red (Life Technologies), then incubated in a shaking incubator at ˚C for 4 hours, simulating egestion of parasites into mammalian hosts and stimulating EV production. Parasites were then separated from the vesicle-enriched supernatant using centrifugation at 300 x g for 5 minutes, then at 2000 x g for 10 minutes. The supernatant was filtered sequentially through a 0.45 μM syringe filter followed by a 0.20 μM syringe filter and centrifuged for 1 hour at 100,000 x g at 4°C using a Beckman Coulter Optima XPN-90™ ultracentrifuge and a SW32.Ti swinging rotor with open-top thin wall polypropylene tubes (16 x 102 mm; Beckman Coulter™, Brea, CA, USA). The supernatant was discarded, and pellets were collected, pooled together, and resuspended in exosome buffer (137 mM NaCl, 20 mM Hepes pH 7.5), before being centrifuged for 1 hour at 100,000 x g at 4 ˚C. The final exosome pellet was resuspended in exosome buffer in approximately 200-300 μL and stored at -80 ˚C.

Protein concentrations of the extracted samples were assessed using the microBCA Protein Assay kit according to manufacturer`s instructions (Thermo Scientific, catalogue number 23235). Particle size distribution and concentration of EV preparations were assessed by nanoparticle tracking analysis using an LM-10 Nanosight machine in the laboratory of Dr. Janus Rak at the Research Institute of the McGill University Health Centre, as previously described (3, 20). A dosage of 10 μg of *Leishmania* EVs/Exosomes was selected for *in vivo* and *in vitro* experiments following established protocols (10).

### Chemicals

Chemical agonists/antagonists used in this study include the selective FPR2 antagonist WRW4 or WRWWWW (1μm; Tocris Biosciences, Ellisville, MO, USA), and the FPR agonist WKYMVm (1μm; Tocris Biosciences, Ellisville, MO, USA).

### Animals and ethics

Male C57BL/6 mice were used in all experiments, and ANXA1-deficient mice were generously provided by Dr. Maziar Divangahi (McGill University) (16). Animal experiments were carried out in containment level 2 pathogen-free housing facilities in the Research Institute of the McGill University Health Center (RI-MUHC). Experiments were performed in accordance with the regulations of the Canadian Council of Animal Care Guidelines (CCAC), and McGill University Animal Care Committee (UACC) under ethics protocol numbers 7791 and 4859. Mice were housed socially in 3–5 mice per IVC cage, with food, water, and soft bedding, and were euthanized after 8–10 weeks using isoflurane and CO_2_ asphyxiation followed by cervical dislocation. Adult male C57BL/6 (6–8 weeks old) mice purchased from Charles River Laboratories (Wilmington, MA, USA) were used for all experiments.

### Murine footpad infections

Groups of five male C57BL/6 mice were infected in the right hind footpad with 5 x 10^6^ stationary-phase *Leishmania major* stationary phase promastigotes, either alone or with 10 μg of *Leishmania* EVs/Exosomes. Ten, 20, or 30 mice were used per experiment, separated between 2, 4, or 6 groups, as described in figure captions. Footpad swelling was measured weekly or bi-weekly with a metric caliper to monitor lesion development, with uninfected footpads used as a negative control. No randomization or blinding was used, and mice in each group were housed in the same cage for the duration of the experiment. Lesion progression was monitored for ten weeks, at the end of which footpads were processed using a limiting dilution assay to determine the parasite burden for select experiments. Mice were euthanized after ten weeks using isoflurane and CO_2_ asphyxiation followed by cervical dislocation.

### Murine intraperitoneal inoculation

Mice (6–8 weeks old, male) were injected intraperitoneally with either endotoxin-free PBS (Wisent Inc, St-Bruno, QC), or 10^8^ stationary phase *L. major* promastigotes, either alone or co-inoculated with *Leishmania* EVs/Exosomes (10 μg). All injections were prepared using endotoxin-free PBS (Wisent Inc, St-Bruno, QC) with a final volume of 250 μL per mouse. Three independent experiments were performed for each experimental design. Six hours post-intraperitoneal infection, mice were sacrificed and generously sprayed with 75% ethanol. The peritoneum was exposed, and 5 ml of cold endotoxin-free PBS (Wisent Inc, St- Bruno, QC, Canada) was injected into the peritoneal cavity, avoiding organ perforation. After injection, the peritoneum was gently massaged to loosen the attached cells into the PBS solution, and lavages were collected by moving the needle in the peritoneal cavity while aspirating. Samples were kept on ice, and the number of live cells in the lavages was counted using a hemocytometer and an optical microscope.

Harvested cells were prepared for microscopy using the Cytospin 4 cytocentrifuge (Thermo Scientific, Waltham, MA, USA). Cells were fixed and stained using the Differential Quik (Diff-Quik) stain kit (Ral Diagnostics, Martillac, France). The percentage and the total number of cell types found in the lavage were counted, along with the percentage of cells infected and the number of *Leishmania* amastigotes found within the cells. The peritoneal lavages were then used for *in vitro* culture and centrifugation to pellet the recruited cells and obtain cell-free supernatants for further analysis.

### *In vitro* culture of myeloid cells

A volume of 200μl of peritoneal lavages was plated in 4-well chamber slides and supplemented with Dulbecco’s modified eagle medium (Wisent, St-Bruno, QC, Canada) with 10% FBS and 1% penicillin-streptomycin-glutamine. Cells were kept at 37 ˚C with 5% CO_2_ for 24–48 hours. Cells were then fixed and stained using Diff-Quik, and a minimum of 200 cells per slide were counted to obtain the percentage of infected cells and number of amastigotes per cell.

### Multiplex cytokine/chemokine quantification assay

A volume of 200μl of the lavage supernatant was analyzed by a multiplex mouse cytokine array/chemokine array 44-plex assay (Eve Technologies, Calgary, AB, Canada), including eotaxin, Erythropoietin, 6Ckine, Fractalkine, G-CSF, GM-CSF, IFNB1, IFN-γ, IL-1α, IL-1β, IL-2, IL-3, IL-4, IL- 5, IL-6, IL-7, IL-9, IL-10, IL-11, IL-12 (p40), IL-12 (p70), IL-13, IL-15, IL-16, IL-17, IL-20, IP- 10, KC, LIF, LIX, MCP-1, MCP-5, M-CSF, MDC, MIG, MIP-1α, MIP-1β, MIP-2, MIP-3α, MIP-3B, RANTES, TARC, TIMP-1, TNF-α, and VEGF. Multiplex laser bead technology was employed, utilizing antibodies coupled to color-coded polystyrene beads, permitting quantification using lasers that excite the fluorescent conjugates. Cytokines and chemokines from the lavage fluid were quantified using data provided by Eve technologies.

### Cell isolation and preparation for immunophenotyping

The right popliteal lymph node was isolated using mechanical disruption through a 70 μM strainer in a complete RPMI medium. Footpads were collected and digested in RPMI 1640 with 5% FBS (Wisent, Saint-Bruno, QC) containing collagenase D (0.5 mg/mL) in the presence of DNAse I (0.005 uM) (Sigma-Aldrich) for 60 minutes at 37 ˚C, then passed through a 70 μm cell strainer. Cells were counted using Trypan Blue (Gibco Thermo Fisher Scientific, Waltham, MA) and kept in complete RPMI medium. For cytokine analysis, 5 x 10^5^ cells were plated in a 96 flat-well culture dish and exposed to Phorbol 12-myristate 13-acetate (PMA; Sigma-Aldrich), ionomycin (Sigma-Aldrich), and monensin (BD Golgi-Stop™, BD Biosciences) following manufacturer’s guidelines for 3 hours before staining.

### Flow cytometry

Single-cell suspensions obtained from cell isolation were stained with the following fluorescence-conjugated monoclonal antibodies purchased from Thermo Fisher Scientific (Waltham, MA) unless otherwise stated: α-CD3 FITC (17A2), α-CD4–Alexa700 (GK1.5), α-CD8-V500 (53-6.7) (BD Biosciences). Intracellular stains included α-Foxp3-FITC or PECy7 (FJK-16s), α-Ki67-BUV395 (B56, BD Biosciences), α-IL17A-APC (eBio17B7), α-IFNγ-BUV737 (XMG1.2, BD Biosciences), and α-IL4-PE (11B11) using the Ebioscience™ Foxp3 staining kit (Thermo Fisher Scientific). Non-viable cells were excluded using fixable viability dye eFluor 780 reagents (Thermo Fisher Scientific). Data were acquired using a FACS Fortessa X-20 flow cytometer (BD Biosciences) and analyzed using FlowJo version 10 software (TreeStar, BD Biosciences).

### Statistical analysis

Statistical significance between groups was determined using unpaired Student *t*-tests, corrected for multiple comparisons using the Holm-Sidak method. Brown-Forsythe and Welch ANOVA tests, with Games-Howell’s correction for multiple comparisons, or two-way ANOVA with multiple comparisons by uncorrected Fisher LSD’s Test or Holm-Sidak tests, were used when comparing multiple groups. P-values lower than 0.05 were considered significant. *P < 0.05, **P < 0.01, ***P < 0.001, and ****P < 0.0001.

### Limiting dilution assay

Limiting dilution assays were performed on footpad lesions at ten weeks post-infection by processing infected tissue and diluting it in PBS. After counting parasites in the extraction solution, 20-fold serial dilutions were prepared in SDM supplemented with 10% FBS. A volume of 100 μL of each dilution was transferred into wells of a microtiter plate. A control plate contained serial dilutions of *in vitro*-cultured *L. major* promastigotes. After seven days of incubation at 26 ˚C, the number of positive wells (presence of motile parasites) and negative wells (absence of motile parasites) was identified by direct observation under an inverted light microscope, enabling the total number of parasites in footpad lesions to be estimated.

## Results

### ANXA1 is required for leishmanial exosome-mediated hyperpathogenesis during experimental *L. major* infection

**Figure 1:**
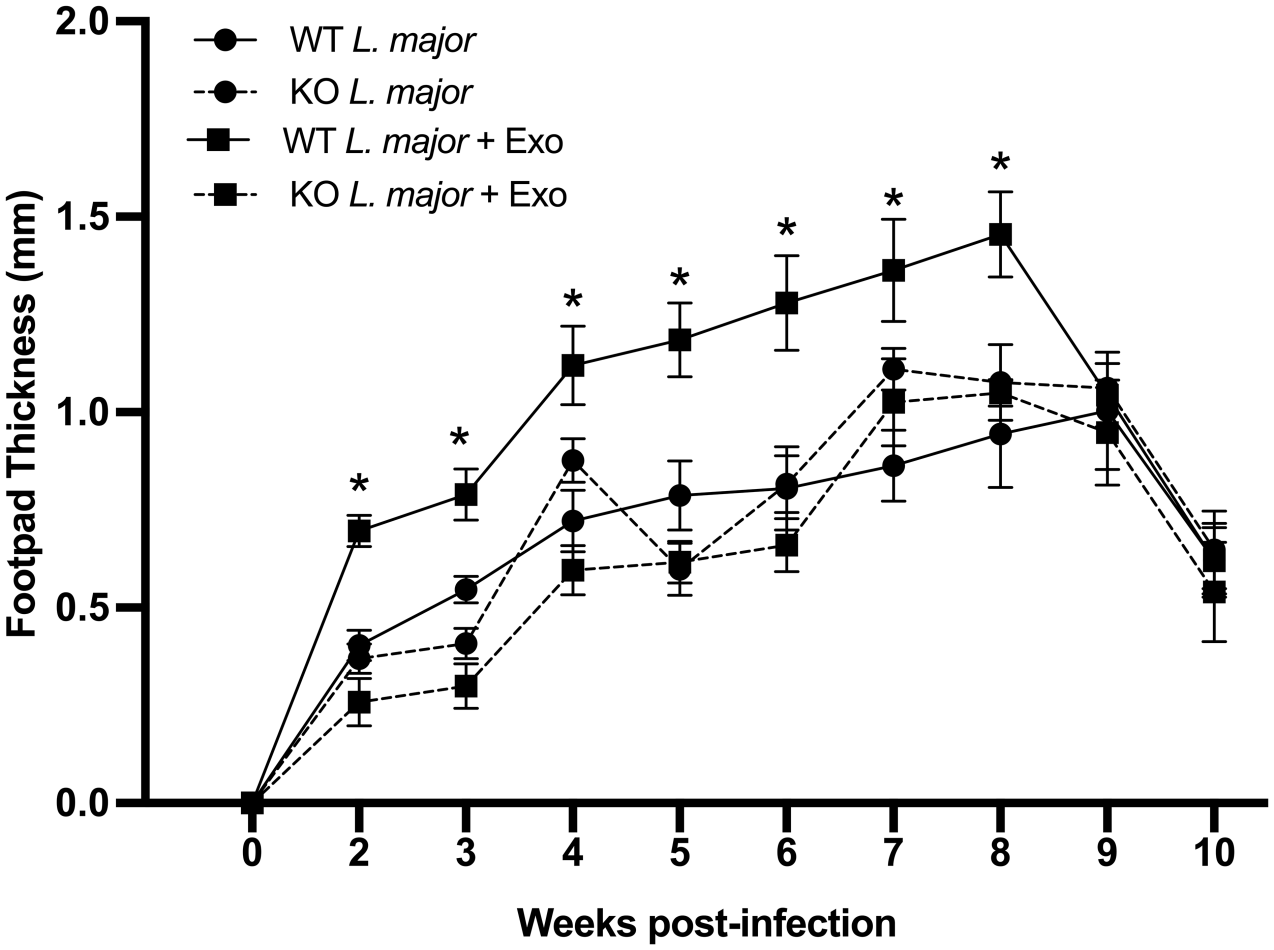
ANXA1 is required for leishmanial exosome-mediated immunopathogenesis during experimental *L. major* infection. *L. major* promastigotes were injected alone or in combination with LeishEXO into footpads of wildtype or ANXA1^-/-^ mice. Lesion thickness was monitored weekly for ten weeks post-infection. Data are represented as mean ± SEM, *n* = 9. Differences were found to be significant using two-way ANOVA with Holm–Sidak’s correction. *P ≤ 0.05, **P ≤ 0.01, ***P ≤ 0.001, ****P ≤ 0.0001, ns, non-significant.

Given the primary constituents of exosomes are lipids and phospholipids, which directly interact with negative-charged phospholipid-sensing annexin receptors, we hypothesized that ANXA1 was involved in the exacerbation of leishmaniasis by leishmanial exosomes (LeishEXO). To study this, we used C57BL/6 mice, which are genetically resistant to leishmaniasis, and mount a strong Th1 response against the parasite (21, 22). In a model of experimental leishmaniasis, footpad infections of wildtype and ANXA1^-/-^ mice were performed using *L. major* either alone or in combination with LeishEXO. The purity of *L. major* exosome preparations was validated (**Supplementary Figure S1**), and the selected dose was 10 μg per mouse – an amount that we have previously reported to elicit an optimal response (10) and that corresponds to approximately 10 (12) particles (23). Strikingly, while wildtype mice responded to the co-inoculation of parasites and exosomes with the expected infective phenotype, mice lacking ANXA1 did not display increased footpad swelling compared to groups infected with parasites alone (**Figure 1**). These findings suggested that ANXA1 was required in the exosome-mediated exacerbation of experimental cutaneous leishmaniasis. This pronounced variance between co-inoculated wildtype and knockout mice was sustained over most of the course of infection, raising additional questions surrounding the effect of ANXA1 deficiency on the adaptive immune response.

### ANXA1 deficiency does not prohibit the generation of a cellular T_H_1 response during experimental *L. major* infection

An efficacious host response against *L. major* requires the polarization of CD4^+^ T helper 1 (T_H_1) cells, and the activation and migration of CD8^+^ T cells, which are critical for parasitic control and clearance (24). Inversely, a T helper 2 (T_H_2)-skewed response has been shown to enable persistent *Leishmania* infection (25, 26). To determine whether the absence of ANXA1 exerted an effect on the development of a productive leishmanicidal adaptive immune response, we sought to characterize immune cell populations and adaptive phenotypes over the course of experimental *L. major* infection.

T cells from the draining popliteal lymph node, a critical site for T cell differentiation, were assessed at various time points post-infection to measure immune polarization (27). Given data indicating that the frequency of IFN-γ^+^ CD4^+^ T cells peaked at week 5 post-infection, indicating that the T_H_1 response was established, we chose this final timepoint for subsequent experiments (**Supplementary Figure S2**).

**Figure 2:**
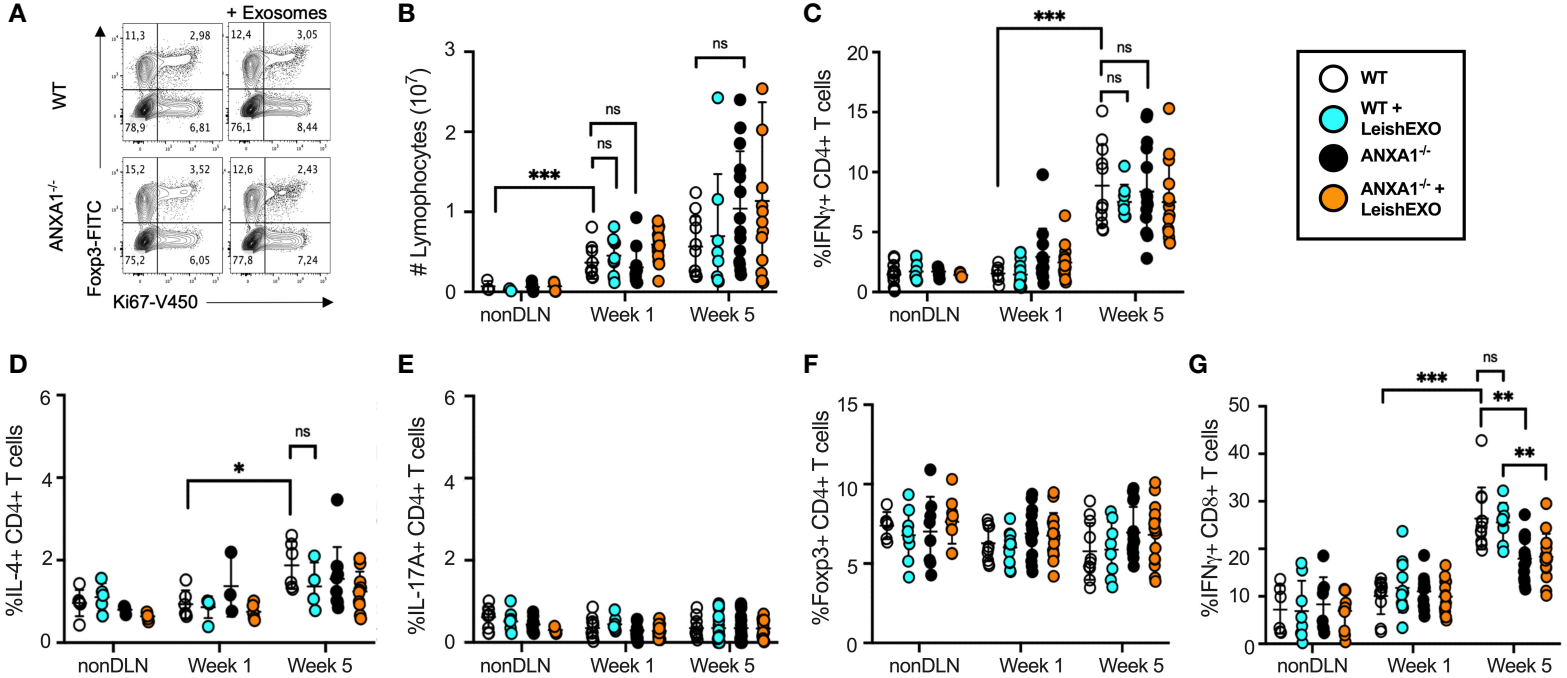
ANXA1 deficiency does not prohibit the generation of a cellular T_H_1 response during experimental *L. major* infection. *L. major* promastigotes were injected alone or in combination with LeishEXO into footpads of wildtype or ANXA1^-/-^ mice. Mice were sacrificed at weeks 1 and 5 post-infection (See also **Supplementary Figure S2**, **Supplementary Figure S3** and **Supplementary Figure S4**), and T cells were isolated from draining (DLN) and non-draining (nonDLN) popliteal lymph nodes. **(A)** Representative dot plot of Foxp3+ and Ki67+ CD3+CD4+ T cells from the DLN at week 5 post-infection. **(B)** Total lymphocytes collected from nonDLN and DLN at weeks 1 and 5 post-infection. Frequency of CD4+ T cells that are **(C)** IFN-γ-producing, **(D)** IL-4-producing, **(E)** IL-17A-producing, **(F)** Foxp3+, and of **(G)** IFN-γ-producing CD8+ T cells collected from nonDLN and DLN at weeks 1 and 5 post-infection. Data are represented as mean ± SEM, *n* = 3. Differences were found to be significant using two-way ANOVA with Holm–Sidak’s correction. *P ≤ 0.05, **P ≤ 0.01, ***P ≤ 0.001, ****P ≤ 0.0001, ns, non-significant.

When comparing T cell populations isolated from wildtype mice comparatively to their ANXA1-deficient counterparts following inoculation with *L. major* alone or in combination with leishmanial exosomes, no differences in the frequency of actively dividing Ki67^+^ T cells were observed between any groups (**Figure 2A**). Further, no statistical difference was observed in the total number of lymphocytes in the popliteal lymph nodes at weeks 1 or 5 between the groups (**Figure 2B**). As expected, IFN-γ- but not IL-4- or IL17A-producing T cells increased significantly in the popliteal lymph node of all groups over the course of infection as the adaptive response was established (**Figures 2C–E**). Extended data showed no difference in the mean fluorescence intensity (MFI) of IFN-γ-producing CD4^+^ T cells between the groups at weeks 1 or 5 (**Supplementary Figure S3A**). The abundance of Foxp3^+^ T_REG_ cells did not vary over the course of infection, suggesting that the immune response was geared towards an effective clearance of the parasite in all groups (24) (**Figure 2F**). Interestingly, when measuring the frequency of IFN-γ-producing CD8^+^ T cells, which have been suggested to have a protective role during *Leishmania* infection (28), a clear increase was observed over time (**Figure 2G**). While this was independent of the presence of exosomes in the initial inoculum, a lower proportion of these cells was identified in knockout mice comparatively to wildtype controls, revealing a potential defect in the generation or retention of IFN-γ-producing CD8^+^ T cells associated with the absence of ANXA1. Notably, while there were no significant differences in the MFI of IFN-γ-producing CD8^+^ T cells at 1 week post-infection, wildtype mice infected with *L. major* alone or in combination with LeishEXO exhibited higher IFN-γ levels than ANXA1-deficient mice infected with the parasite alone at the 5 week time point (**Supplementary Figure S3B**). These findings agree with previous work indicating that ANXA1 is implicated in, but not critical to, the generation of a T_H_1 response (29). However, this difference likely exerts a limited effect considering our earlier footpad infection model demonstrated that both wildtype and knockout mice displayed similar footpad swelling response to parasites alone (**Figure 1**).

Additional studies assessing the phenotypes of cells derived from the infected footpads showed no difference in the abundance of CD4^+,^CD8^+,^ and γδ T cells in knockout mice by week 1 (**Supplementary Figures 4A-C**). Further, the co-inoculation of LeishEXO induced a significant increase in the frequency of CD11b^+^ cells in footpads of wildtype mice, suggesting that dendritic cells or macrophages were primarily affected by the presence of *Leishmania-*derived EVs (**Supplementary Figure S2D**). Though this trend was also observed in cell populations derived from ANXA1-deficient mice, it was not statistically significant.

Collectively, these observations indicate that the ANXA1-dependent effect of leishmanial exosomes does not significantly influence the generation of a protective adaptive immune response at later time points post-infection. This suggests that ANXA1 may alter the innate immune response during promastigote implantation and early inflammatory events.

### ANXA1 deficiency does not affect *Leishmania/*LeishEXO-mediated cellular and humoral inflammation during acute experimental *L. major* infection

**Figure 3:**
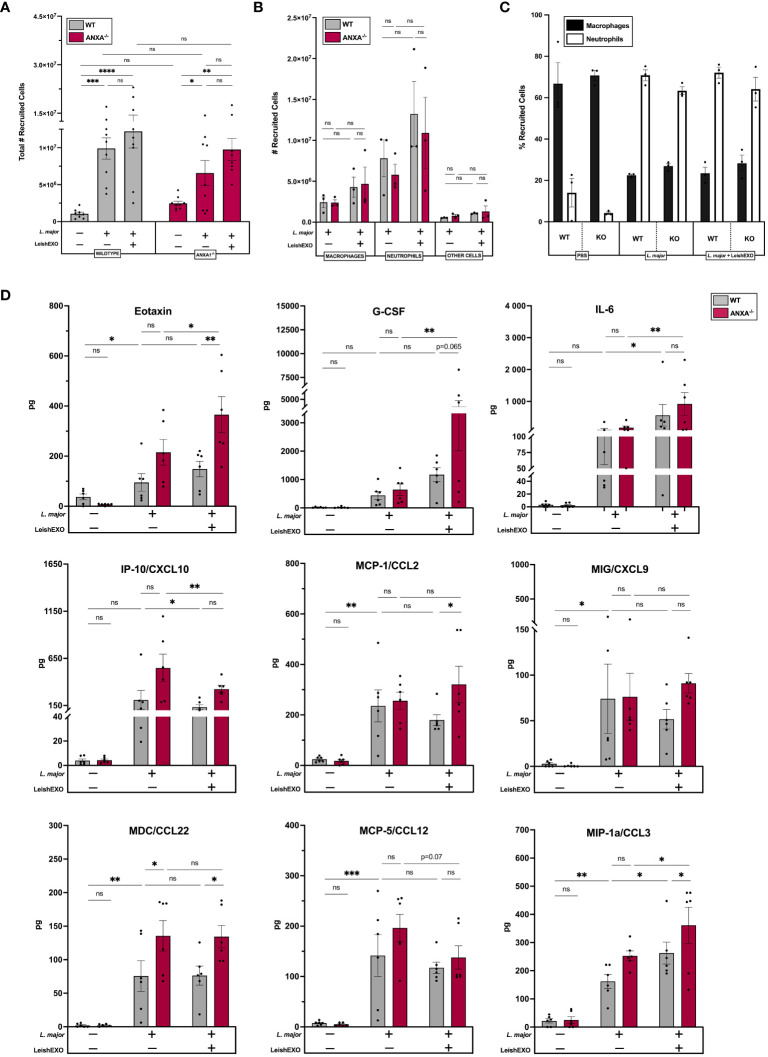
ANXA1 deficiency does not affect *Leishmania/*LeishEXO-mediated inflammatory cell recruitment or production of inflammatory mediators during acute experimental *L. major* infection. Wildtype or ANXA1^-/-^ mice were injected intraperitoneally with *L. major* promastigotes, alone or in combination with LeishEXO for 6 hours. **(A)** Total recruited inflammatory cells, **(B)** total macrophages, neutrophils, or other cells recruited to the peritoneum, and **(C)** percentage of neutrophils and macrophages within recruited inflammatory cells. **(D)** Protein expression of inflammatory mediators in peritoneal lavage fluid (See also **Supplementary Figure S5**). Data are represented as mean ± SEM, **(A–C)** *n* = 3 and **(D)** *n* = 6. Differences were found to be significant using two-way ANOVA with Holm–Sidak’s correction. *P ≤ 0.05, **P ≤ 0.01, ***P ≤ 0.001, ****P ≤ 0.0001, ns, non-significant.

To further decipher ANXA1’s role in the immune response to leishmaniasis, we aimed to characterize its involvement in early inflammatory events following *Leishmania* infection. Peritoneal infections of wildtype and ANXA1^-/-^ mice were performed using *L. major* alone or in combination with LeishEXO, following a well-established model of acute infection and inflammatory cell recruitment (30). Cells recruited to the peritoneal cavity were counted 6 hours post-infection, revealing an increase in total recruited cells in all infected groups, independent of the addition of LeishEXO (**Figure 3A**). Further, no significant differences were observed between total recruited cells in wildtype and ANXA1^-/-^ mice.

When further characterizing myeloid cell populations recruited to the peritoneal cavity, similar numbers of macrophages and neutrophils were identified in wildtype and ANXA1^-/-^ mice (**Figure 3B**). While a trend indicated a slight increase in myeloid cell recruitment when *Leishmania* infection was accompanied with LeishEXO, this difference was not statistically significant. This observation was confirmed using the percentage of total recruited inflammatory cells that were macrophages and neutrophils, indicating that both *L. major* alone and in combination with leishmanial exosomes recruited more neutrophils than macrophages comparatively to a PBS control (**Figure 3C**). Similar percentages were observed in wildtype and ANXA1^-/-^ mice. Together, these data suggest that ANXA1-deficiency plays a limited role in inflammatory cell recruitment during acute *L. major* infection.

We next sought to measure the effects of ANXA1 deficiency and of LeishEXO stimulation on the expression of inflammatory mediators to further characterize their role in early inflammatory events during *Leishmania* infection. The production of cytokines and chemokines was assessed using a 44-multiplex assay that measured levels of protein in the peritoneal lavage, revealing that eotaxin, G-CSF, IL-6, IP-10 (CXCL10), MCP-1/CCL2, MIG/CXCL9, MDC/CCL22, MCP-5/CCL12, and MIP-1a/CCL3 displayed notable differences between groups (**Figure 3D**). Levels of eotaxin, a potent chemoattractant for eosinophils (31), were elevated in both wildtype and ANXA1^-/-^ mice infected with *L. major* alone. Further, co-inoculation with LeishEXO exhibited significantly increased eotaxin levels in ANXA1-deficient mice compared to their wildtype counterparts – a finding that was not observed in response to *L. major* infection alone. The peritoneal accumulation of G-CSF, a potent stimulator of granulocyte development in the bone marrow (31), was significantly increased by the addition of LeishEXO to the inoculum in ANXA1-deficient mice only. While a trend indicated that ANXA1^-/-^ mice produced more G-CSF than their wildtype counterparts when stimulated with both *L. major* and LeishEXO, this finding was not statistically significant. Levels of IL-6, an essential player in the initiation of the inflammatory response (31), were shown to be significantly enhanced by the addition of LeishEXO to the *L. major* infection in both wildtype and knockout mice – though no difference in IL-6 accumulation was observed between the animals. Interestingly, levels of IP-10/CXCL10, a chemokine involved in Th1 polarization (31), decreased in both wildtype and ANXA1-deficient mice when inoculated with *L. major* and LeishEXO compared to mice infected with the parasite alone. Further, levels of the inflammatory chemokine MCP-1/CCL2 (31) were similarly increased in both models following *L. major* infection, although protein expression levels were much higher in ANXA1^-/-^ mice than their wildtype counterparts when co-inoculated with LeishEXO. This same trend was observed in the peritoneal accumulation of the chemokine MIG/CXCL9, although not to statistically significant levels. When assessing levels of MDC/CCL22, involved in monocyte migration (31), protein accumulation was notably higher in ANXA1-deficient mice than in wildtype mice, in groups either having received *L. major* alone or in combination with LeishEXO (**Figure 3D**). The addition of LeishEXO to the *L. major* inoculum did not further enhance MDC/CCL22 protein expression. Levels of MCP-5/CCL12 detected in the peritoneal cavity followed a similar trend, although not to statistically significant levels. Finally, co-inoculation of LeishEXO with *L. major* significantly increased levels of the chemokine MIP-1a/CCL3 in both ANXA1^-/-^ and wildtype mice comparatively to groups having received the parasite alone. Further, MIP-1a/CCL3 levels were higher in ANXA1-deficient mice than in their wildtype counterparts when inoculated with *L. major*/LeishEXO – a trend that was similarly observed in mice receiving *L. major* alone, though not statistically significant. Additional inflammatory mediators implicated in a robust leishmanicidal response, including IL-1β, IL-10, IL-12 (p70), IL-17, and TNF-α, did not display notable changes in response to the co-inoculation of LeishEXO or ANXA1 deficiency (**Supplementary Figure S5**). Altogether, these results suggest that ANXA1 deficiency does not affect inflammatory cell recruitment and only moderately affects inflammatory cytokine and chemokine production in acute *L. major* infection.

### ANXA1 deficiency abrogates LeishEXO-mediated myeloid cell infection

**Figure 4:**
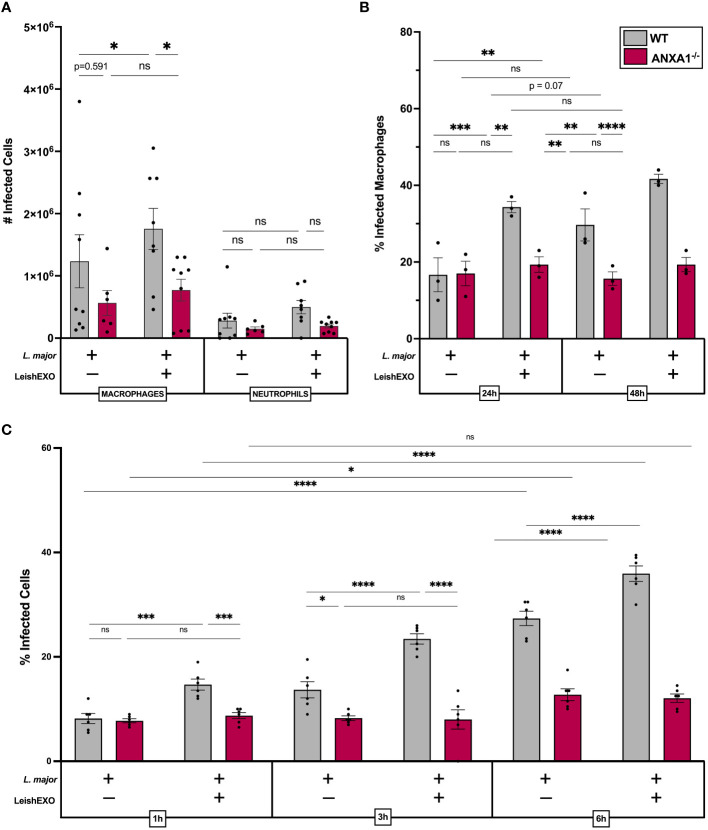
ANXA1 deficiency abrogates LeishEXO-mediated myeloid cell infection during acute experimental *L. major* infection. Wildtype or ANXA1^-/-^ mice were injected intraperitoneally with *L. major* promastigotes, alone or in combination with LeishEXO for 6 hours. **(A)** Total infected myeloid cells in peritoneal lavage. **(B)** Percentage of infected macrophages derived from peritoneal lavage following 24–48 hours of *in vitro* culture. **(C)** Percentage of infected naïve peritoneal macrophages inoculated with *L. major* promastigotes alone or in combination with LeishEXO for 1, 3, or 6 hours. Data are represented as mean ± SEM, **(A)** *n* = 9, **(B)** *n* = 3, and **(C)** *n* = 6. Differences were found to be significant using two-way ANOVA with Holm–Sidak’s correction. *P ≤ 0.05, **P ≤ 0.01, ***P ≤ 0.001, ****P ≤ 0.0001, ns, non-significant.

We next aimed to address whether parasitemia and myeloid cell infection varied between wildtype and knockout mice. Infection rates of cells isolated from the peritoneal cavity were counted, revealing that the co-inoculation of LeishEXO in wildtype mice led to a significant increase in the number of infected neutrophils, which was not observed in their ANXA1-deficient counterparts (**Figure 4A**). Peritoneal macrophage infection, which initially showed no difference between wildtype and knockout animals 6 hours post-infection, was further dissected using an *ex vivo* culture model to account for potential longer required incubation times. Macrophages were harvested from infected ANXA1^-/-^ and wildtype mice peritoneal lavages and cultured for 24–48 hours before counting (**Figure 4B**). The percentage of infected macrophages derived from wildtype mice increased over time and was significantly higher when animals received both *L. major* and LeishEXO. Interestingly, the proportion of infected macrophages derived from ANXA1-deficient mice was independent of LeishEXO co-inoculation and did not increase over time.

To corroborate these findings, we aimed to decipher earlier time points post-stimulation in an *in vitro* model, using naïve cultured peritoneal macrophages isolated from both animal models. Cell cultures were inoculated with *L. major* alone or in combination with LeishEXO, and infection was counted over a short-term time course experiment (**Figure 4C**). At 1, 3, and 6 hours post-infection, co-inoculation of LeishEXO resulted in significantly higher percentages of infected wildtype macrophages. This effect was absent in macrophages derived from ANXA1-deficient mice, which displayed a constant percentage of infected cells across all time points and experimental groups. Overall, these data indicate that ANXA1 deficiency significantly abrogates myeloid cell infection by *L. major*, which is typically exacerbated by the presence of LeishEXO in wildtype animals.

### FPR2 blockade inhibits LeishEXO-mediated hyperinfective *L. major* infection

**Figure 5:**
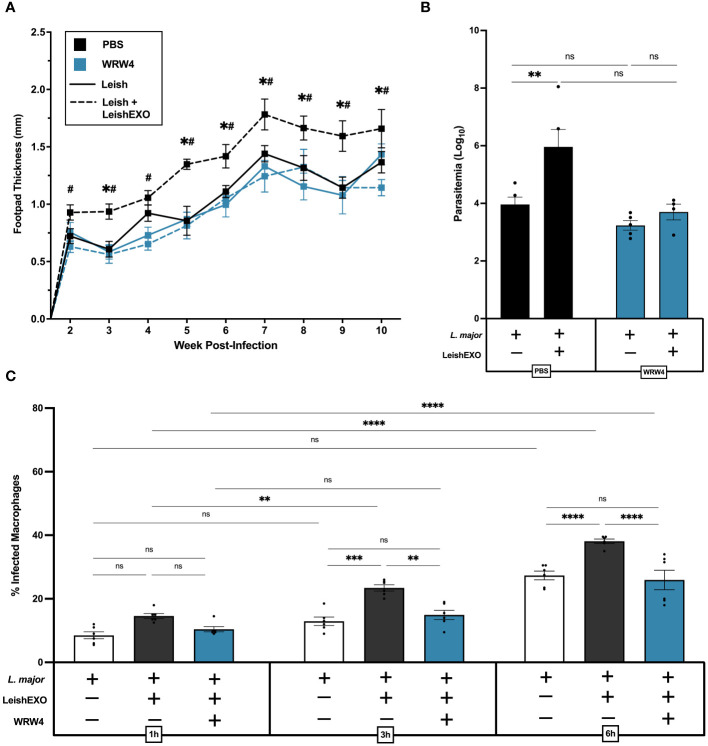
FPR2 blockade with antagonist WRW4 inhibits LeishEXO-mediated immunopathogenesis of *L. major* infection. *L. major* promastigotes were injected alone or in combination with LeishEXO into footpads of mice pre-treated with WRW4 or PBS. **(A)** Lesion thickness was monitored weekly for ten weeks post-infection. **(B)** Footpad parasitemia at 10 weeks post-infection. **(C)** Percentage of infected naïve peritoneal macrophages pre-treated with WRW4 or left untreated, inoculated with *L. major* promastigotes, alone or in combination with LeishEXO, for 1, 3, or 6 hours (See also **Supplementary Figure S6**). Data are represented as mean ± SEM, **(A, B)** *n* = 9 and **(C)** *n* = 6. Differences were found to be significant using two-way ANOVA with Holm–Sidak’s correction. *P ≤ 0.05, **P ≤ 0.01, ***P ≤ 0.001, ****P ≤ 0.0001, ns = non-significant. All significant (P ≤ 0.05) differences in footpad thickness are indicated for PBS-treated *L. major* vs. PBS-treated *L. major*/LeishEXO (#) and WRW4-treated *L. major/*LeishEXO vs. PBS-treated *L. major*/LeishEXO (+).

We next sought to understand the mechanisms underlying ANXA1’s involvement in the LeishEXO-mediated infectivity of *L. major* to myeloid cells and associated cutaneous pathology. FPR2 is a G-protein coupled receptor (GPCR) expressed by myeloid cells (32), among many other cell types, which binds to the cleaved form of ANXA1. As FPR2 is an established downstream receptor of ANXA1, we aimed to assess the implication of the ANXA1/FPR2 receptor-ligand interaction in an *in vivo* model of experimental cutaneous leishmaniasis. Wildtype C57BL/6 mice were treated with the FPR2 antagonist WRW4 (1 µM) or PBS 30 minutes prior to infection with *L. major* alone or in combination with LeishEXO. While footpads of control mice developed the expected inflammatory phenotype when co-inoculated with *L. major* and LeishEXO, for which footpad thickness far exceeded that observed in mice infected with *L. major* alone, WRW4 pre-treated mice did not display enhanced LeishEXO-mediated pathology (**Figure 5A**). This unresponsiveness to LeishEXO when pre-treated with WRW4, displaying similar footpad inflammation in both *L. major* and *L. major*/LeishEXO infected groups, implicated FPR2 as a key player in LeishEXO-mediated pathogenesis. Measuring the parasite burden in footpads at 10 weeks post-infection with a limiting dilution assay indicated that, while PBS-treated control mice displayed higher parasitemia when inoculated with *L. major* in combination with LeishEXO than when infected with the parasite alone, this was not the case in WRW4 pre-treated mice (**Figure 5B**). The similar parasite burden measured in WRW4 pre-treated mice, independent of the presence of LeishEXO in the initial inoculum, further corroborated that FPR2 is involved in exosome-mediated pathogenesis of *Leishmania*.

An *in vitro* peritoneal macrophage infection model was then used to validate the effect of WRW4 on the blockade of LeishEXO-mediated pathogenesis. Experimental groups included cells infected with *L. major* alone, co-inoculated with *L. major* and LeishEXO, or pre-treated with WRW4 followed by infection with *L. major*/LeishEXO (**Figure 5C**). In untreated cells, the addition of LeishEXO to the *L. major* inoculum induced a significantly higher proportion of infected macrophages at 3 and 6 hours post-infection – a trend that was visible as early as 1 hour post-infection, though not to statistically significant levels. Strikingly, cells pre-treated with WRW4 prior to the *L. major/*LeishEXO challenge displayed similar infection levels to untreated macrophages infected with *L. major* alone, indicating an unresponsiveness to LeishEXO. Interestingly, despite higher levels of infection, numbers of amastigotes per cell remained constant across all experimental groups (**Supplementary Figure S6**). Thus, these data show that the FPR2 antagonist WRW4 effectively inhibits a ligand-receptor interaction that is necessary for LeishEXO-mediated exacerbation of *L. major* pathogenesis. Altogether, this suggests that the ANXA1/FPR2 interaction in myeloid cells is a key contributor to skin pathology and parasitemia during cutaneous leishmaniasis.

### FPR agonism enhances *L. major* infection independently of ANXA1 and LeishEXO

**Figure 6:**
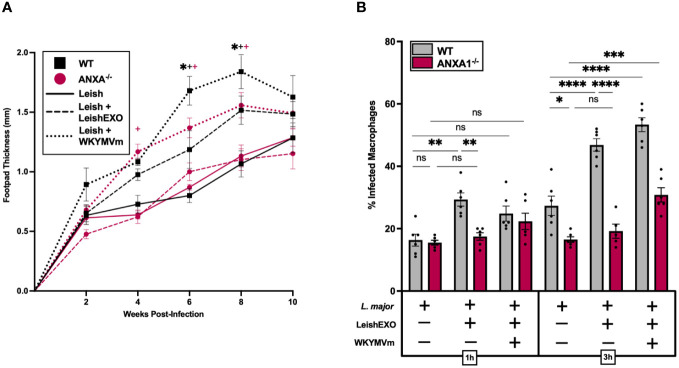
FPR agonism with WKYMVm enhances hyperinfective *L. major* infection independently of ANXA1 and LeishEXO. *L. major* promastigotes were injected alone, in combination with LeishEXO, or along with WKYMVm into footpads of wildtype or ANXA1^-/-^ mice. **(A)** Lesion thickness was monitored bi-weekly for ten weeks post-infection. **(B)** Percentage of infected naïve peritoneal macrophages inoculated with *L. major* promastigotes alone, in combination with LeishEXO, or along with WKYMVm, for 1 or 3 hours. Data are represented as mean ± SEM, *n* = 6. Differences were found to be significant using two-way ANOVA with Holm–Sidak’s correction. *P ≤ 0.05, **P ≤ 0.01, ***P ≤ 0.001, ****P ≤ 0.0001, ns = non-significant. All significant (P ≤ 0.05) differences in footpad thickness are indicated for *L. major* vs. *L. major*/LeishEXO (#) and *L. major/*LeishEXO vs. *L. major*/WKYMVm (+).

We next sought to determine whether direct activation of FPRs with the agonist WKYMVm could bypass the deficient LeishEXO/ANXA1 interaction in ANXA1^-/-^ mice and result in a similar phenotype to that of *L. major*/LeishEXO co-inoculation in our wildtype *in vivo* model. To address this, wildtype and ANXA1-deficient C57BL/6 mice were infected with *L. major* alone, *L. major* in combination with LeishEXO, or *L. major* along with WKYMVm (**Figure 6A**). As expected, co-inoculation of *L. major* and LeishEXO resulted in significantly enhanced footpad swelling in wildtype mice comparatively to the parasite alone, though the severity of cutaneous pathology was surpassed by the group receiving *L. major* and WKYMVm. Strikingly, ANXA1-deficient mice, while unresponsive to the additive effects of LeishEXO inoculation along with *L. major*, displayed a hyperinfective phenotype and greater footpad swelling when *L. major* was co-inoculated with WKYMVm. This increase in footpad thickness indicates that the FPR agonist was capable of bypassing ANXA1 deficiency, directly stimulating the cellular receptor and downstream pathogenesis.

An *in vitro* study of the effect of FPR agonism on myeloid cell infection by *L. major* was then performed using peritoneal macrophages isolated from both wildtype and ANXA1^-/-^ mice. Cells received either *L. major* alone, in combination with LeishEXO, or along with WKYMVm, and infection rates were measured at 1 and 3 hours post-inoculation (**Figure 6B**). The addition of the FPR agonist led to an increase in the percentage of infected ANXA-deficient macrophages at 3 hours post-infection, albeit less pronounced than the effect observed in cells derived from wildtype mice. This significant increase in infected cells, particularly when compared to the unresponsiveness to LeishEXO, provides further evidence that activation of FPRs with WKYMVm bypasses the deficient ANXA1 signaling cascade that is implicated in LeishEXO recognition. Together, these data highlight the importance of FPRs in *Leishmania*-associated cutaneous pathology and indicate that stimulation of the ANXA1/FPR axis by LeishEXO is likely implicated in this process.

## Discussion

Though exosomes produced by the protozoan parasite *Leishmania* are well-established drivers of virulence (3, 10, 12), mechanisms underlying their capacity to promote infection remain elusive. Our group has previously described that co-inoculation of *L. major* with leishmanial exosomes (LeishEXO) in a murine footpad model induces the development of cutaneous lesions far more severe than that caused by the parasite alone (33). In the present study, experiments using ANXA1-deficient C57BL/6 mice demonstrated that Annexin A1 (ANXA1) – a crucial player in various physiological and pathological processes (34–36) – is involved to this LeishEXO-mediated response.

ANXA1’s involvement in the adaptive immune response remains poorly described, with conflicting studies indicating that it can mediate both pro-inflammatory and anti-inflammatory responses (37–39). Also known as lipocortin, ANXA1 is constitutively produced by T cells (40, 41)and has been implicated in lymphocyte proliferation, differentiation, and activation (37–39). When ascertaining whether ANXA1 deficiency hindered the development of a Th1 response in a Th1-biased *L. major* murine infection model, we observed limited to no effect on the development of effector T cell subsets, including Th1 (IFNγ+ CD4+), Th2 (IL-4+ CD4+), Th17 (Il-17A+ CD4+), or regulatory (Foxp3+CD4+) T cells. Whereas some previous studies indicate that ANXA1 deficiency favors the development of Th2 T cells in a murine model of KLH-stimulation (42), others have observed an increase in Th1-associated inflammatory mediators of allergic inflammation (37, 43), suggesting that this process is antigen-specific. Further, most of these studies utilize autoimmune and allergy models (38) which cannot properly recapitulate the immunopathogenesis of infectious agents. While ANXA1-deficiency has been associated with higher T cell proliferation *in vitro* (42), we found similar expression levels of the proliferative marker Ki67 in populations derived from both wildtype and knockout mice. In addition, no variation in total lymphocyte counts was observed in the knockout model comparatively to its wildtype counterpart, indicating that ANXA1^-/-^ does not inhibit T cell homing to the lymph node. However, functional studies of these subsets may be of interest, given evidence that ANXA1-deficient T cells exhibit impaired responses to TCR stimulation (42), along with transcriptomic studies, to conclusively determine that these knockout and wildtype lymphocyte populations are identical. Further, while B cells express particularly low levels of ANXA1/FPR2 (37) and their role in leishmaniasis is limited (44), immunophenotyping these populations may uncover differences in the adaptive immune response.

Variations were noted, however, when assessing the frequency and MFI of IFNγ-producing CD8+ T cells, which contribute to the generation of the Th1 response and are particularly important in the resistance against primary and secondary *L. major* infection (28). Higher frequencies of these cells in wildtype mice indicate a potential immune deficiency caused by the absence of ANXA1. Further, while infected footpads from both wildtype and ANXA1^-/-^ mice displayed similar T cell populations, larger populations of Cd11b+ myeloid cells were identified in wildtype mice, suggesting innate immune involvement. Thus, given limited variations in adaptive immune profiles between wildtype and ANXA1-deficient mice, we determined that the innate immune response was likely responsible for the important differences observed in LeishEXO-mediated lesion pathology.

The role of ANXA1 is much better described in innate immune cascades, whereby its interaction with FPR2 leads to the recruitment of and differentiation of monocytes, and neutrophil apoptosis (39) – inflammatory events that recapitulate the “Trojan Horse” immunopathogenic mechanism of early *Leishmania* infection (2). To evaluate whether ANXA1^-/-^ mice therefore exhibited variations in myeloid cell population recruitment compared to wildtype mice, we utilized an acute peritoneal *L. major* infection model. While infection and recruitment of entire myeloid cell populations in the footpad would be a particularly interesting way to study this, infiltrates at early time points post-infection are notoriously difficult to isolate due to limited accessibility of small cell populations during the retrieval process (45). In fact, very few studies assess innate inflammatory infiltrates in murine footpads and utilize histological sectioning and staining of tissues, which would be of limited use when studying small intracellular pathogens (46). Thus, the acute peritoneal infection model was chosen, given its established use to study the rapid onset of infection and the innate immune response (30) – which would not be as evident in a footpad model – allowing us to decipher early events in myeloid cell recruitment more effectively. Further, peritoneal macrophages display significantly less heterogeneity than tissue-specific populations throughout the footpad (45, 47), facilitating subsequent experimental steps. Interestingly, our data demonstrated that the total number of cells, including macrophages and neutrophils, as well macrophage to neutrophil recruitment ratios, were not significantly affected by the absence of ANXA1 regardless of LeishEXO stimulation – though further phenotypic studies of recruited cells could illuminate phenotypic differences between subpopulations. Given the ANXA1/FPR axis is one of many interactions involved in the recruitment of myeloid cells, many of which are directly upregulated by *Leishmania*, the injection of parasites into the peritoneal cavity likely stimulates multiple cascades at once, overshadowing any ANXA1-specific effect. Further characterization of the peritoneal microenvironment using a cytokine and chemokine multiplex assay of the lavage fluid indicated variations in few inflammatory mediators. Of these, levels of IL-6 and CXCL10 increased in both wildtype and knockout mice in response to LeishEXO co-inoculation, implicating *Leishmania* exosomes in inflammatory and lymphocyte recruitment processes, though these were not reflected in T cell populations. Similarly, despite consistent Treg populations, levels of the chemoattractant CCL22 were notably higher in ANXA1^-/-^ mice regardless of the addition of LeishEXO. Protein expression of eotaxin, G-CSF, and CCL3 displayed an additive effect of ANXA1^-/–^ and LeishEXO-dependent increase, though innate immune cell populations were similarly unaffected. Further, the expression of key cytokines involved in leishmaniasis-mediated inflammation, including IL-1β, IL-10, IL-12 and TNF-α (48), remained constant across all experimental groups. Together, these findings suggest that the addition of LeishEXO to *L. major* inoculum does not significantly alter myeloid cell recruitment or the production of inflammatory mediators during acute *L. major* infection, independently of ANXA1. Given the lack of explicit modulation of the adaptive or innate immune responses by LeishEXO in wildtype or ANXA1^-/-^ mice, we next studied host cells.

Analysis of the infection rate of myeloid cells derived from wildtype or ANXA1-deficient mice by *L. major* indicated a significant LeishEXO-dependent increase in infectivity. The severity of cutaneous leishmaniasis has been shown to be a direct function of the number of internalized parasites during initial infection (49), with higher infection rates persisting over the course of disease. Previous work by our lab has shown that exosomes produced by *Leishmania* are co-egested along with the parasite into a mammalian host during the sandfly vector’s bloodmeal (10). Given that uptake of leishmanial exosomes is extremely rapid (50, 51), and that the half-life of EVs in circulation is in the order of 2–30 minutes (52), LeishEXO-mediated effects must occur immediately following inoculation and cause permanent changes, enabling persistence of the hyperinfective phenotype long after exosomes have degraded. Such would be the case if LeishEXO increased the number of myeloid cells infected at early timepoints post-infection, causing cutaneous pathology to be exacerbated accordingly and maintained over time, without necessarily altering the fate of the adaptive response in the later stages of the infection.

In addition, our data indicates that the LeishEXO-mediated increase in infectivity is dependent on the presence of ANXA1, therefore implicating the ANXA1/FPR axis in this process. ANXA1 is known to bind to and activate both FPR1 and FPR2, leading to various immune cascades which result in either pro-inflammatory or pro-resolving effects depending on ligands and context. Specifically, ANXA1/FPR1 interactions generally mediate wound closure, particularly in the context of bacterial infections (53, 54). Conversely, interaction with the FPR2 receptor is thought to regulate multiple inflammatory, anti-inflammatory, and pro-resolving effects, and exhibits greater sensitivity and responsiveness to ANXA1 (53, 55). Additionally, while less is known about FPR3, it is also a receptor for ANXA1, albeit with significantly less affinity, and may play a role in modulating immune responses (37). Of the FPRs, the interaction of ANXA1 with FPR2 is more extensively studied and documented in the context of inflammation and immune responses (56). Given that ANXA1 deficiency does not prohibit the generation of a cellular T_H_1 response to experimental *L. major* infection, the ANXA1/FPR1 interaction is likely not essential in our model, indicating that the ANXA1/FPR2 axis may be implicated in this immunopathogenic process.

Pre-treatment of wildtype C57BL/6 mice with the FPR2 antagonist WRW4 was capable of inhibiting LeishEXO-mediated immunopathogenesis, decreasing footpad swelling as well as parasitemia. *In vitro* work corroborated these findings, indicating that FPR2 blockade inhibits LeishEXO-mediated enhancement of macrophage infections, exhibiting similar infection rates to cells incubated with *L. major* alone. This is of notable interest, as while it has been put forward as a potential target for influenza infection due to its role in virion internalization and persistence (32, 57), this is the first report of a protozoan parasite exploiting the ANXA1/FPR2 axis to establish greater infection. Additional evidence indicates that the ANXA1/FPR2 interaction promotes endosomal transport (58) – a finding that is particularly interesting given *Leishmania*’s exploitation of endosomes to form parasitophorous vacuoles within host cells. Further, it is tempting to speculate that the therapeutic success FPR2 antagonists have displayed against influenza viruses in preclinical trials could be leveraged in the fight against leishmaniasis (32), although additional pre-clinical and clinical studies are required to assess their efficacy. Additional challenges arise, however, due to heterogenous immunopathogenesis mechanisms of different species of *Leishmania*, as while the antagonist may be beneficial to *L. major* infection, ANXA1 is associated with disease resolution in cutaneous *L. (Viannia) braziliensis* infection (18). Thus, species identification would be required prior to treatment, reducing its potential use in resource-limited settings. Further, the antagonist is not entirely specific to FPR2, exhibiting low affinity for other FPRs (59); thus, the potential influence of FPR1 and FPR3 cannot be entirely ruled out.

Different species of *Leishmania* employ diverse strategies to evade the immune response and establish infection. This can be directly observed through the action of their exosomes, whereby species-specific cargo of proteins, lipids, and nucleic acids may exert distinct effects on the immune system. For instance, while *L. major* exosomes have been shown to induce a pro-inflammatory response in macrophages, exosomes from *L. (V.) braziliensis* have been implicated in promoting an immunosuppressive environment, favoring parasite survival and persistence (11). These differences may explain divergent roles for ANXA1 in the immunopathogenesis of different *Leishmania* species. Thus, while the ANXA1/FPR axis, mediated by leishmanial exosomes, may be involved in greater parasite internalization in the context of *L. major* infection, future studies should assess whether these mechanisms are shared by other *Leishmania* species.

Experiments using the FPR agonist WKYMVm were used to assess whether direct activation of FPRs could bypass the effects of ANXA1 deficiency and replicate the phenotype generated by co-inoculation of LeishEXO in wildtype animals. Strikingly, the addition of WKYMVm to the *L. major* inoculum induced footpad inflammation in ANXA1^-/-^, very closely mirroring that of *L. major*/LeishEXO in their wildtype counterparts, indicating that direct stimulation of FPRs induces a LeishEXO-like phenotype. *In vitro* experiments further corroborated that the agonist was capable of partially rescuing the expected infectivity, although not to levels observed in wildtype animals. Although we were particularly interested in the effects of FPR2 stimulation, WKYMVm acts as a non-specific agonist for FPRs 1, 2, and 3 (59). While our study primarily attributes the observed effects to the interaction between ANXA1 and FPR2, we cannot exclude the possibility that WKYMV may also be acting through these receptors. Further studies using more selective agonists and antagonists for each FPR subtype are needed to delineate the specific contributions of FPR1, FPR2, and FPR3 in the context of LeishEXO’s effects on *L. major* infection. Of additional interest is the observation that, while the number of infected cells increases with LeishEXO/FPR stimulation, the number of parasites within infected myeloid cells is similar between experimental groups, indicating that the primary goal of this LeishEXO-mediated process is to enhance cell entry rather than increase parasite burden. The exposure of host cells to LeishEXO could trigger cellular responses that facilitate the entry of parasites into cells but limit subsequent parasite replication or survival within the cell, resulting in single-cell infection without increasing the overall parasite burden per cell. Additional studies surrounding the dynamics of parasite internalization will be essential to elucidating this process.

Altogether, these data implicate the ANXA1/FPR axis as a potential contributor to LeishEXO-mediated immunopathogenesis during *L. major* infection. Specifically, ANXA1 interacts with FPRs to enhance the recruitment and infection of myeloid cells, leading to increased early infection rates that persist over time and severe cutaneous lesions. Indeed, despite higher infectivity rates, variations in cytokine and chemokine levels suggest that the primary role of ANXA1/FPR in LeishEXO-mediated processes is to enhance cell entry rather than significantly alter the overall inflammatory profile. Given that FPR2 stimulation overlaps with downstream effectors of the T_H_2 response, the ANXA1/FPR2 interaction may promote persistence and chronicity following parasite uptake (56). Additional parasite-derived components are necessary, however, to the development of this phenotype, including the packaging of sufficient levels of the surface metalloprotease GP63 within the EVs, enabling downstream modulation of host cell signaling pathways, including the activation of SHP-1 and dephosphorylation of ERK1/2 (11, 33). Along with the modulation of cytoskeletal organization and of the phagocytic capacity of myeloid cells upon FPR2 activation, the interaction between host and parasite factors may explain, in part, the increase in the initial infection of host cells by *Leishmania*. Further, while the exact mechanisms by which ANXA1 interacts with FPRs are still under investigation, studies suggest that the binding of ANXA1 to lipids, particularly phospholipids, may influence its interaction with FPR2 indirectly (60). This may provide the missing link between *Leishmania* exosomes, which have an outer layer constituted primarily of lipids and phospholipids (33), and the ANXA1/FPR axis.

Moreover, we hypothesize that the parasitic virulence factor GP63, packaged within leishmanial exosomes, may directly contribute to LeishEXO-mediated stimulation of the ANXA1/FPR axis. As a non-specific metalloprotease, GP63 may cleave mammalian ANXA1, thereby enabling its interaction with the FPRs on monocytes, enhancing their recruitment and infection by the parasite (61). Given that it is primarily the cleaved form of ANXA1 that interacts with the receptor (37–39), this may modulate host signaling pathways, leading to greater parasite internalization and contributing to the hyperinfective phenotype and enhanced lesion severity, although additional studies are required to investigate this potential mechanism. This further implicates FRP2 specifically, as it is displays the greatest sensitivity to cleaved ANXA1, and is a promiscuous receptor capable of interacting with a variety of endogenous and exogenous ligands, such as exosome-derived lipids (62).

In summary, this study elucidates the role of the ANXA1/FPR axis in the exacerbation of cutaneous leishmaniasis mediated by leishmanial exosomes, whereby initial infection is enhanced and maintained over the course of infection, resulting in increased severity of disease. Through a comprehensive series of experiments utilizing ANXA1-deficient mice, as well as agonists and antagonists of FPRs, we delineated a potential mechanism underlying this phenomenon, and have provided novel insights into the involvement of ANXA1 in the immunopathogenesis of leishmaniasis, which may be leveraged in the development of therapeutics for leishmaniasis.

## Data availability statement

The original contributions presented in the study are included in the article/**Supplementary materials**. Further inquiries can be directed to the corresponding author.

## Ethics statement

The animal study was approved by McGill University Animal Care Committee (UACC) under ethics protocol numbers 7791 and 4859. The study was conducted in accordance with the local legislation and institutional requirements.

## Author contributions

AdSLF: Conceptualization, Data curation, Formal analysis, Investigation, Methodology, Visualization, Writing – original draft, Writing – review & editing. AL: Investigation, Validation, Visualization, Writing – original draft, Writing – review & editing. FA: Formal analysis, Investigation, Validation, Visualization, Writing – review & editing. CP: Funding acquisition, Resources, Supervision, Writing – review & editing. MO: Conceptualization, Data curation, Funding acquisition, Project administration, Resources, Supervision, Writing – review & editing.
