# Supplementary material for: Implication of the Annexin 1/FPR axis in leishmanial exosome-mediated Leishmania major skin hyperpathogenesis
Source: Front Immunol. 2024 Jul 15;15:1436151. doi: 10.3389/fimmu.2024.1436151 (PMC11284082; doi:10.3389/fimmu.2024.1436151)
Supplement: Supplementary file 1 [file DataSheet_1.docx]

Supplementary Material

**
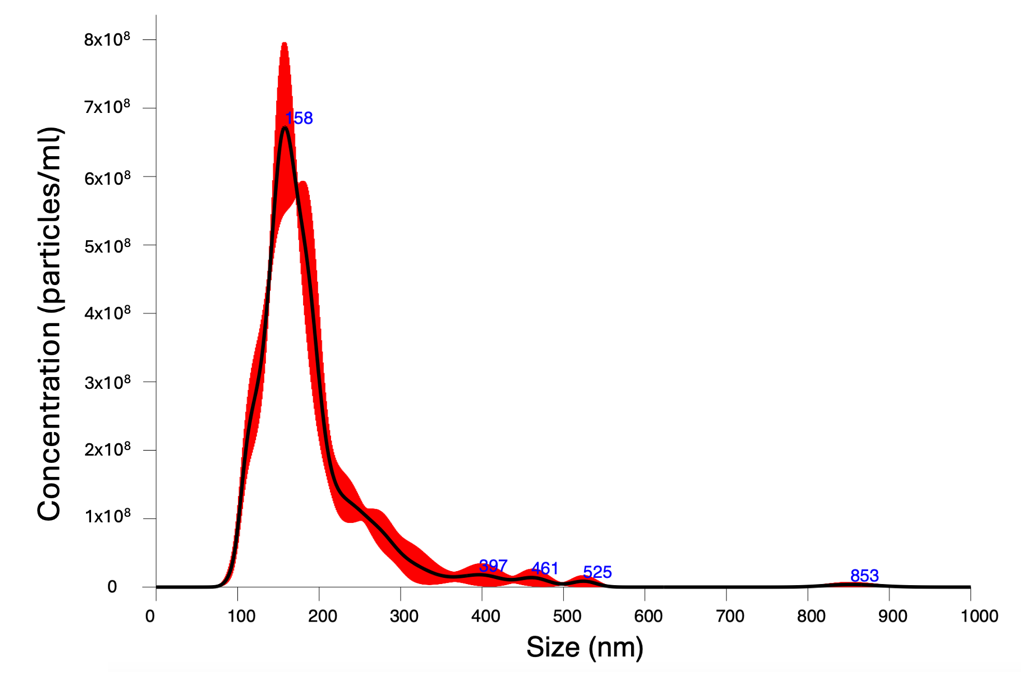
**

**Supplemental Figure 1. *L. major* exosome preparations are comprised of small extracellular vesicles.** *L. major* exosomes were isolated using filtration/ultracentrifugation, and purity was validated by nanoparticle tracking analysis using an LM-10 Nanosight. A representative figure reveals that preparations include a major peak at 158 nm, indicative of exosomes or small extracellular vesicles.

**
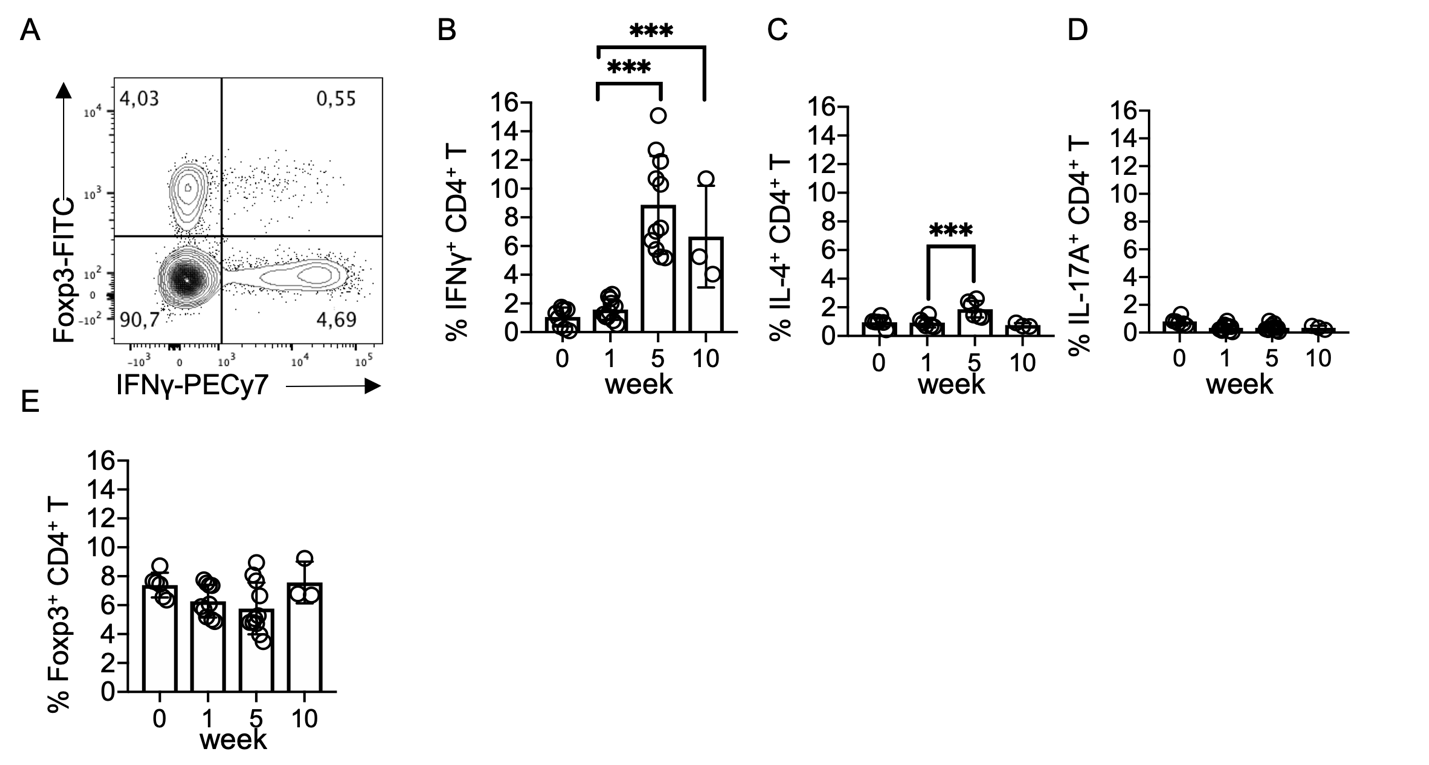
**

**Supplemental Figure 2.** **A Th1 response in the popliteal lymph node is established by week 5 post *Leishmania major* infection.** *L. major* promastigotes were injected into footpads of wildtype mice. Mice were sacrificed at weeks 1, 5, and 10 post-infection, and T cells from draining (DLN) and non-draining (nonDLN) popliteal lymph nodes were isolated and exposed PMA, ionomycin and GolgiStop® for 3 hours. (A) Representative dot plot of FITC-labelled Foxp3+ and PECy7-labelled IFN-γ+ CD4+ T cells from the draining popliteal lymph node at week 5 post-infection. Frequency of (B) IFN-γ-producing CD4+ T cells, (C) IL-4-producing CD4+ T cells, (D) IL-17A-producing CD4+ T cells, and (E) Foxp3+ CD4+ T cells collected from nonDLN and draining lymph nodes at weeks 0, 1, 5, and 10 post-infection. Data are represented as mean ± SEM, *n* = 3. Differences were found to be significant using one-way ANOVA with Tukey’s correction. *P ≤ 0.05, **P ≤ 0.01, ***P ≤ 0.001, ns = non-significant.

**
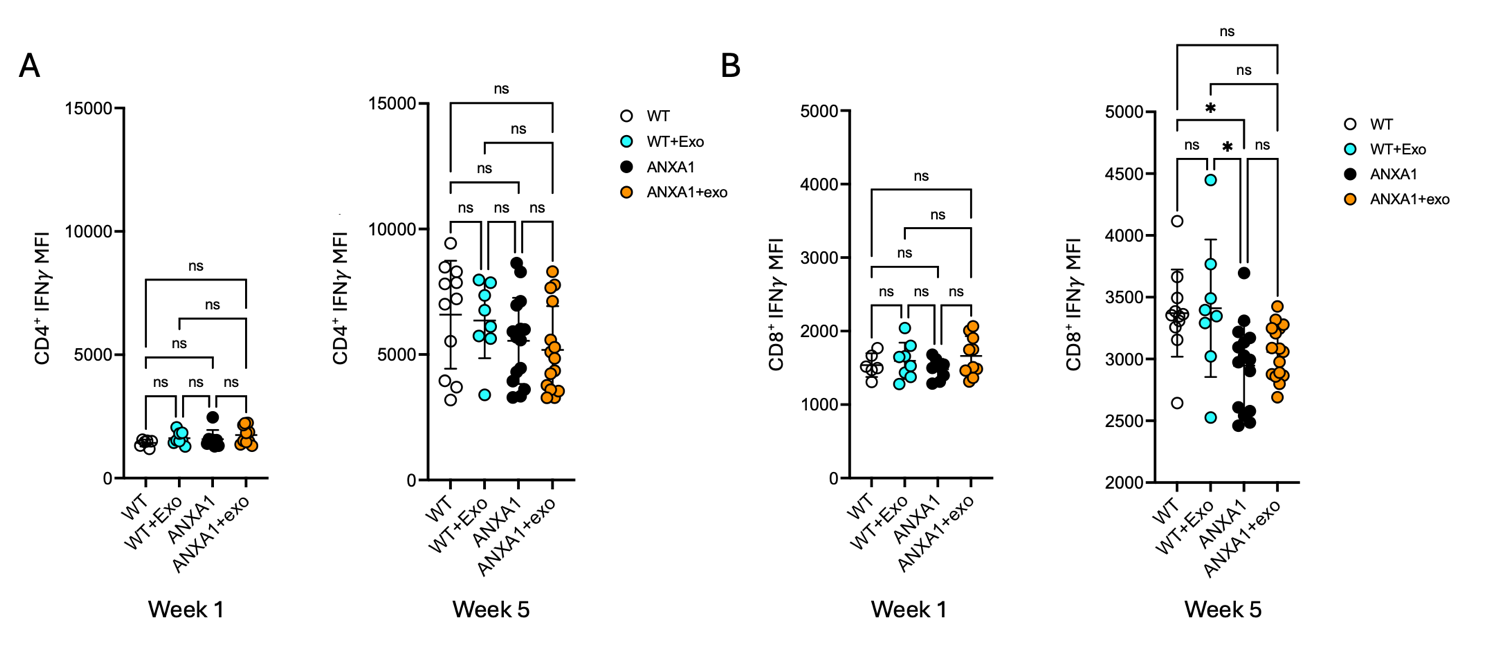
**

**Supplemental Figure 3. *Leishmania major* moderately alters IFN-γ production by CD8+ T cells in ANXA1-deficient mice.** *L. major* promastigotes were injected alone or in combination with LeishEXO into footpads of wildtype or ANXA1^-/-^ mice. Mice were sacrificed at weeks 1 and 5 post-infection, and T cells were isolated from draining and non-draining popliteal lymph nodes were isolated and exposed PMA, ionomycin and GolgiStop® for 3 hours. Mean fluorescence intensity (MFI) of IFN-γ-producing (A) CD4^+^ and of (B) CD8^+^ T cells was measured weeks 1 and 5 post-infection. Data are represented as mean ± SEM, *n* = 3. Differences were found to be significant using two-way ANOVA with Holm–Sidak’s correction. *P ≤ 0.05, **P ≤ 0.01, ***P ≤ 0.001, **** ≤ 0.0001, ns = non-significant.

**
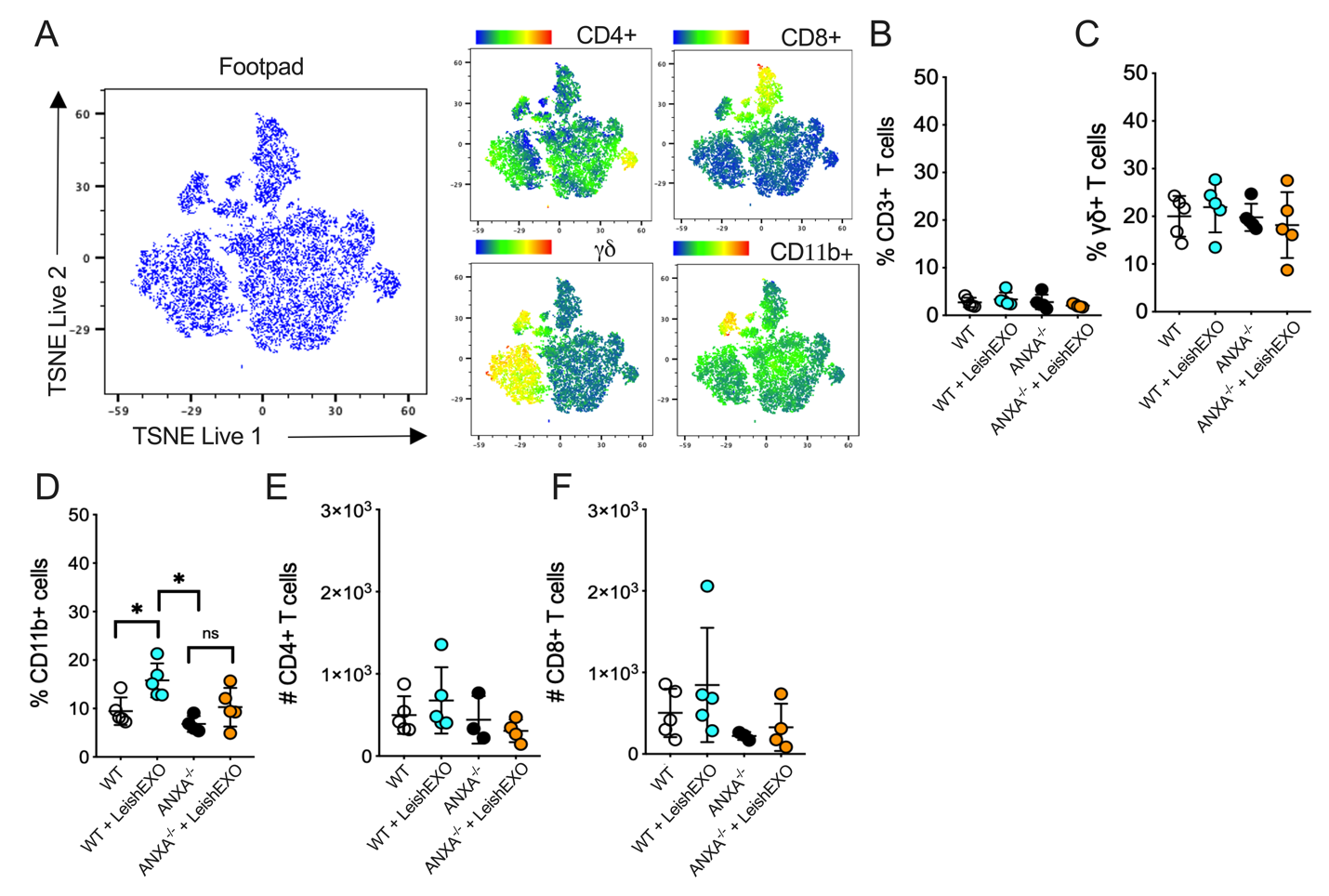
**

**Supplemental Figure 4. *Leishmania major*-derived EVs promote the early accumulation of CD11b+ cells rather than γδ or αβ T cells in the footpad.** *L. major* promastigotes were injected into footpads of wildtype mice. Mice were sacrificed one week following infection, and cells from the right footpad were isolated and analyzed by flow cytometry. (A) TSNE analysis of total live cells isolated from the right footpad. Compiled 2D representation of CD4, CD8, γδ TCR and CD11b expression among populations. Frequency of (A) CD3+ cells, (B) γδ T cells, and (C) CD11b+ cells among live cells. Number of (E) CD3+ CD4+ T cells and (F) CD3+ CD8+ T cells. Data are represented as mean ± SEM, *n* = 3**–**5. Differences were found to be significant using one-way ANOVA with Tukey’s correction. *P ≤ 0.05, **P ≤ 0.01, ***P ≤ 0.001, ns = non-significant.

**
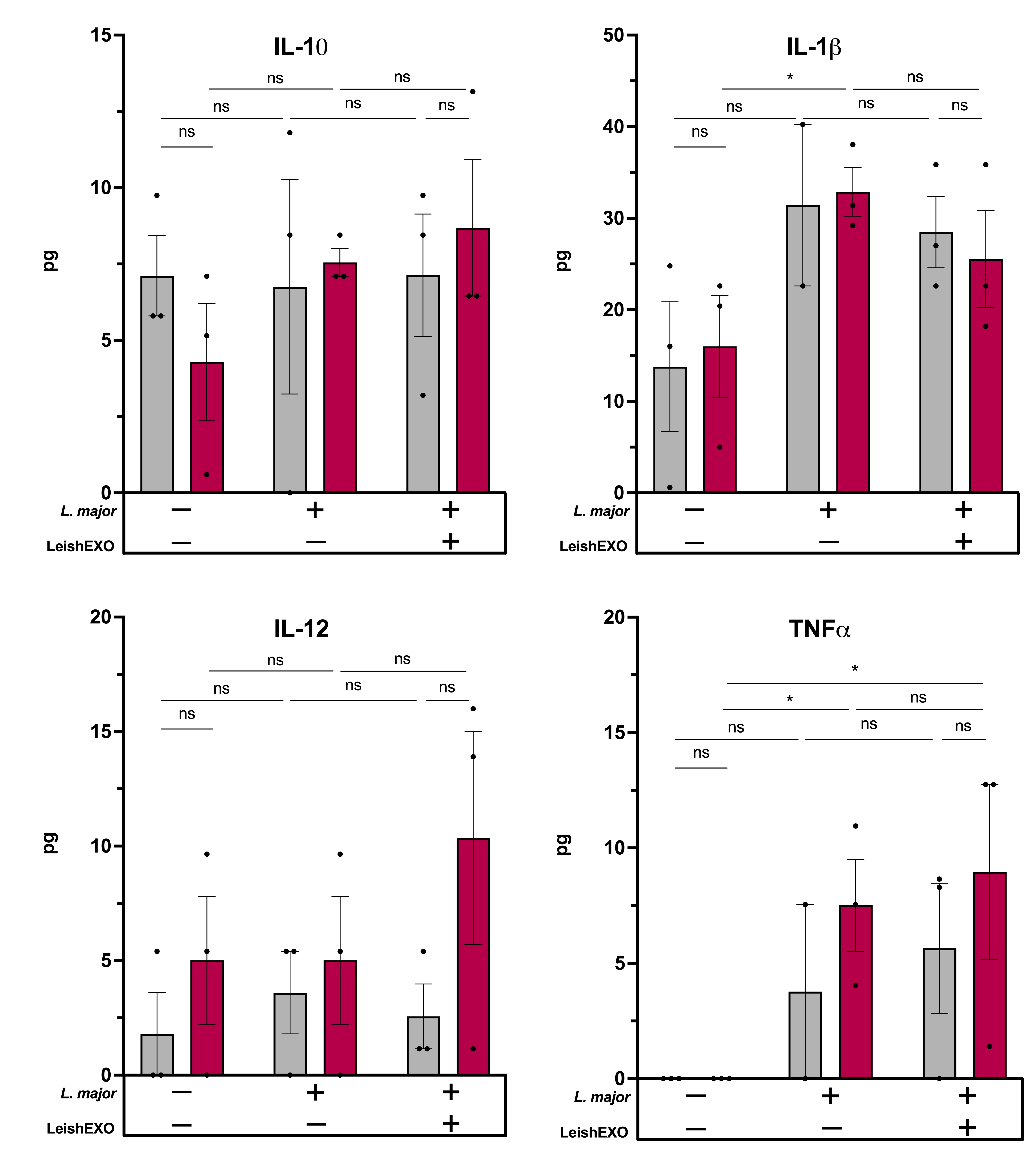
**

**Supplemental Figure 5. ANXA1 deficiency exerts a limited effect on *Leishmania/*LeishEXO-mediated inflammatory cytokine/chemokine production.** Wildtype or ANXA1^-/-^ mice were injected intraperitoneally with *L. major* promastigotes, alone or in combination with LeishEXO. Mice were sacrificed 6 hours post-infection and peritoneal lavage was performed with endotoxin-free PBS. Inflammatory mediator protein expression was measured using a multiplex cytokine/chemokine quantification assay of lavage fluid. Data are represented as mean ± SEM, *n* = 6. Differences were found to be significant using two-way ANOVA with multiple comparisons by uncorrected Fisher’s LSD. *P ≤ 0.05, **P ≤ 0.01, ***P ≤ 0.001, ns = non-significant.

**

**

**Supplemental Figure 6. LeishEXO stimulation does not affect the number of parasites infecting myeloid cells.** Naïve peritoneal macrophages were isolated from wildtype mice, pre-treated with WRW4 or left untreated, then infected with *L. major* promastigotes, alone or in combination with LeishEXO, for 1, 3, or 6 hours before counting numbers of amastigotes per cell. Data are represented as mean ± SEM, *n* = 3. Differences were found to be significant using two-way ANOVA with Holm–Sidak’s correction. *P ≤ 0.05, **P ≤ 0.01, ***P ≤ 0.001, **** ≤ 0.0001, ns = non-significant.
